# Assessing consciousness in patients with locked-in syndrome using their EEG

**DOI:** 10.3389/fnins.2025.1604173

**Published:** 2025-09-11

**Authors:** Sophie Adama, Martin Bogdan

**Affiliations:** ^1^Department of Neuromorphic Information Processing, Leipzig University, Leipzig, Germany; ^2^Center for Scalable Data Analytics and Artificial Intelligence (ScaDS.AI), TU Dresden and Leipzig University, Leipzig, Germany; ^3^School of Embedded Composite Artificial Intelligence (SECAI), Leipzig, Germany

**Keywords:** Brain-Computer Interface, complete locked-in syndrome, complexity, connectivity, consciousness, EEG, frequency, soft clustering

## Abstract

Research indicates that locked-in syndrome (LIS) patients retain both consciousness and cognitive functions, despite their inability to perform voluntary muscle movements or communicate. Brain-Computer Interfaces (BCIs) provide a means for these patients to communicate, which is crucial, as the ability to interact with their environment has been shown to significantly enhance their wellbeing and quality of life. This paper presents an innovative approach to analyzing electroencephalogram (EEG) data from four LIS patients to assess their consciousness levels, referred to as normalized consciousness levels (NCL) in this study. It consists of extracting different features based on frequency, complexity, and connectivity measures to maximize the probability of correctly determining the patients' actual states given the inexistence of ground truth. The consciousness levels derived from this approach aim to improve our understanding of the patients' condition, which is vital in order to build effective communication systems. Despite considerable inter-patient variability, the findings indicate that the approach is effective in detecting neural markers of consciousness and in differentiating between states across the majority of patients. By accurately assessing consciousness, this research aims to improve diagnosis in addition to determining the optimal time to initiate communication with these non-communicative patients. It is important to note that consciousness is a complex and difficult concept to define. In this study, the term “consciousness level” does not refer to a medical definition. Instead, it represents a scale of NCL values ranging from 0 to 1 representing the likelihood of the patient being fully conscious (1) or not (0).

## 1 Introduction

Locked-In Syndrome (LIS) is a rare but clinically significant neurological disorder that poses substantial challenges for both patients and caregivers. It is most often caused by damage to the ventral region of the pons in the brainstem, although injuries to the midbrain or bilateral internal capsules have also been reported (Gazzaniga et al., 2018; Parker and Parker, 2004; Laureys et al., 2005). The condition may result from stroke, traumatic brain injury (TBI) or progressive neurological diseases such as Amyotrophic Lateral Sclerosis (ALS) and Guillain-Barré syndrome (Kübler et al., 2001; Kübler, 2020).

The American Congress of Rehabilitation Medicine (ACRM) defines LIS based on several clinical features: persistent eye opening, aphonia or markedly reduced speech, severe paralysis affecting all four limbs (quadriplegia or quadriparesis), and preserved cognitive function (NARIC, 1995). Communication is typically limited to vertical eye movements or blinking (Schnakers et al., 2009). While patients usually retain normal sleep-wake cycles (Gazzaniga et al., 2018), chronic insomnia can develop as the condition progresses (Soekadar et al., 2013; Posner et al., 2007). LIS is extremely uncommon, affecting an estimated one in 20,000 individuals.

There is currently no definitive cure. However, medications such as riluzole, which may protect motor neurons by limiting glutamate-induced excitotoxicity, can slightly delay disease progression in ALS-related cases (NINDS, 2013; Bear et al., 2016; Parker and Parker, 2004). In ALS, initial symptoms include muscle weakness and wasting, which over time lead to the loss of all voluntary movement, including speaking, swallowing, and breathing. This progression typically results in death from respiratory failure within five years of onset (Murguialday et al., 2011).

Brain-Computer Interfaces (BCIs) could offer a solution enabling such patients to engage with their environment. When there are no residual muscle movement left after transitioning into a complete LIS (CLIS) state, it becomes impossible to ascertain if the patients are conscious or not at a specific time point. Thus, there are no ground-truth for consciousness for CLIS patients. This makes it extremely challenging to identify the optimal time to communicate with them, particularly given the limited research focused on evaluating patients' consciousness. As we are focusing on EEG in this study, the following subsections only include research in communication and consciousness assessment including LIS and/or CLIS patients using non-invasive EEG or ECoG.

### 1.1 BCI-based communication for CLIS patients

The successful implantation of neurotrophic electrodes in humans, as reported in Kennedy and Bakay (1998), marked a foundational step in the development of BCI systems aimed at enabling communication for individuals with severe motor impairments. This study confirmed the technical feasibility of long-term neural signal acquisition and demonstrated promising results for potential BCI applications.

The first speller for LIS patients with ALS, developed by Birbaumer et al. in the late 1990s, employed self-regulated Slow Cortical Potentials (SCPs) to control a device that allowed patients to select letters on a screen (Birbaumer et al., 1999; Hinterberger et al., 2003). This system evolved into a Thought Translation Device (TTD), enabling the selection of words and pictograms, and was tested successfully with five LIS patients (Birbaumer et al., 2000). Further developments included an adapted web browser and email interface, called NESSI, which allowed patients to interact with web pages using SCPs (Bensch et al., 2007).

SCP-based BCIs are slow, with brain responses taking around five seconds, and the training can cause patient fatigue (Kübler et al., 2001). Faster brain responses, such as the P300, have been explored. For example, Oken et al. implemented a rapid serial visual presentation (RSVP) paradigm with nine LIS patients, finding that while all could complete easier tasks, only one could manage the most difficult (Oken et al., 2014). Steady State Visual Evoked Potential (SSVEP)-based systems have also been used, showing less mental workload compared to P300 systems, but these systems are impractical for patients without gaze control and can still cause fatigue (Lesenfants et al., 2014; Combaz et al., 2013).

Auditory P300-based BCIs were developed for visually impaired LIS patients, but reliable communication using these systems has been difficult to achieve Kübler et al. (2009). In some cases, invasive techniques like electrocorticography (ECoG) have been used successfully, such as in a study where an ALS patient achieved high spelling accuracy with an implanted system (Vansteensel et al., 2016).

Attempts to communicate with CLIS patients initially failed, raising questions about transferring brain control learned in LIS to CLIS (Kübler and Birbaumer, 2008). However, later research reported successful communication with CLIS patients using EEG-based BCIs, with a vibro-tactile P300 and motor imagery, achieving high accuracies of up to 90% (Guger et al., 2017). Another study involved a CLIS patient using an intracortical microelectrode array to spell words by modulating neural firing rates (Chaudhary et al., 2022). The patient was able to effectively convey his needs and perspectives, proving that it is possible to communicate with CLIS patients using their brain signals.

These studies often do not assess patients' consciousness before the communication tasks, although following commands could indicate consciousness (Lesenfants et al., 2015). Assessing this capability beforehand might be more logical so to know when to communicate with them.

### 1.2 Consciousness assessment in LIS and CLIS patients

Correct diagnosis is critical for optimizing patient care, especially for enabling communication with loved ones despite severe impairments. Most studies assessing consciousness after TBI focus on patients with Minimally Conscious State (MCS) or Unresponsive Wakefulness Syndrome (UWS), with few including LIS patients due to the rarity of the condition. This section addresses studies involving at least one LIS or CLIS patient.

#### 1.2.1 Consciousness assessment using Brain Sensory Responses

Sensory stimuli (auditory, tactile, visual) are often used to assess consciousness by eliciting event-related potentials (ERPs) such as P300, which is commonly triggered by auditory stimuli (Sergent et al., 2017; Annen et al., 2020). In one study, P300 responses were used to differentiate between patients with UWS, MCS, and LIS (Perrin et al., 2006). Results indicated that while P300 was present in all LIS patients, it was absent in two UWS patients. Similarly, another study showed that P300 responses predicted recovery in patients, including two LIS patients, after 12 months (Zhang et al., 2017).

Other methods, such as hybrid visual BCI combining P300 and SSVEP, have shown that LIS patients can follow commands by focusing on specific visual stimuli (Pan et al., 2014). Vibro-tactile P300 paradigms have also been effective, with 5 out of 6 LIS patients successfully eliciting P300 responses (Lugo et al., 2014). Additionally, motor imagery tasks have been used to assess awareness, with spectral analysis of EEG revealing task-dependent changes in LIS and MCS patients (Goldfine et al., 2011).

#### 1.2.2 Consciousness assessment using resting state data

Resting state data, which captures spontaneous neural activity without requiring task performance, is another method for assessing consciousness. This approach often uses spectral analysis, connectivity measures, and complexity metrics like Lempel-Ziv complexity (LZC) (Jain and Ramakrishnan, 2020). In one study, Perturbational Complexity Index (PCI) derived from LZC was used to distinguish MCS from UWS patients, with PCI values in LIS patients comparable to healthy controls (Gosseries et al., 2014). Spectral connectivity has also been employed to assess consciousness, revealing strong brain network connectivity in patients misdiagnosed as unresponsive (Chennu et al., 2017).

Few studies have focused on LIS / CLIS patients using resting state data. However, preliminary research using measures like coherency and entropy showed potential for detecting consciousness in CLIS patients, though these methods typically consider only one or two signal features at a time (Adama et al., 2019, 2021).

### 1.3 Motivation and research objective

This paper presents a study assessing LIS patients' consciousness using EEG data recorded over several years from four patients during an EOG-based communication. A previously successful approach in estimating consciousness levels in one CLIS and patients with disorders of consciousness (Adama and Bogdan, 2023a,b) was applied. To maximize accuracy in determining consciousness, multiple features are extracted from pre-processed EEG signals to capture various aspects of conscious states. By integrating these features, this approach enhances the likelihood of accurately assessing the patients' true state, especially in the absence of a definitive ground truth. The obtained results were subsequently compared with the patients' performance accuracy to evaluate the method's performance. Consciousness is difficult to define, with various interpretations across different fields. In this study, the term "consciousness level" does not adhere to the medical definition. Instead, it refers to a normalized scale, where 0 represents unconsciousness and 1 indicates full consciousness. This scale, designated as normalized consciousness level (NCL) in the context of this research, is used to quantify and compare states of awareness rather than provide a clinical diagnosis.

The NCL method was developed to help identify when patients with CLIS are conscious, so that attempts at communication can be made at the right time. The approach could potentially be used to assist in diagnosis, but its main focus is on finding the best moment to connect with the patient. This is especially important in CLIS, where patients cannot move at all, and traditional methods that rely on movement or speech are no longer useful. Common clinical tools like the Coma Recovery Scale-Revised (CRS-R) (Giacino et al., 2004) and the ALS Functional Rating Scale-Revised (ALSFRS-R) (Cedarbaum et al., 1999) serve very different purposes. CRS-R is meant to assess disorders of consciousness, such as the vegetative state or minimally conscious state. However, LIS / CLIS are not disorders of consciousness. These patients are often fully aware but unable to respond. CRS-R depends heavily on a patients ability to show signs of awareness through movement or speech (Giacino et al., 2004), which is not possible with LIS/CLIS patients, especially in late stages.

The ALSFRS-R, on the other hand, is used to measure physical decline in ALS, including speech, mobility, and breathing. But it says nothing about whether the person is conscious. In people with advanced ALS, scores often drop to very low levels even if the patient is fully aware (Cedarbaum et al., 1999; Tonin et al., 2020). This makes both CRS-R and ALSFRS-R unsuitable for measuring consciousness in this type of patients.

The paper is organized as follows: the EEG data acquisition protocols, experimental setups as well as a description of the approach used is introduced in Section 2. The results of the analysis are presented in Section 3 and discussed, before concluding in Section 4.

## 2 Methods

### 2.1 Patients and data description

**Figure 1:**
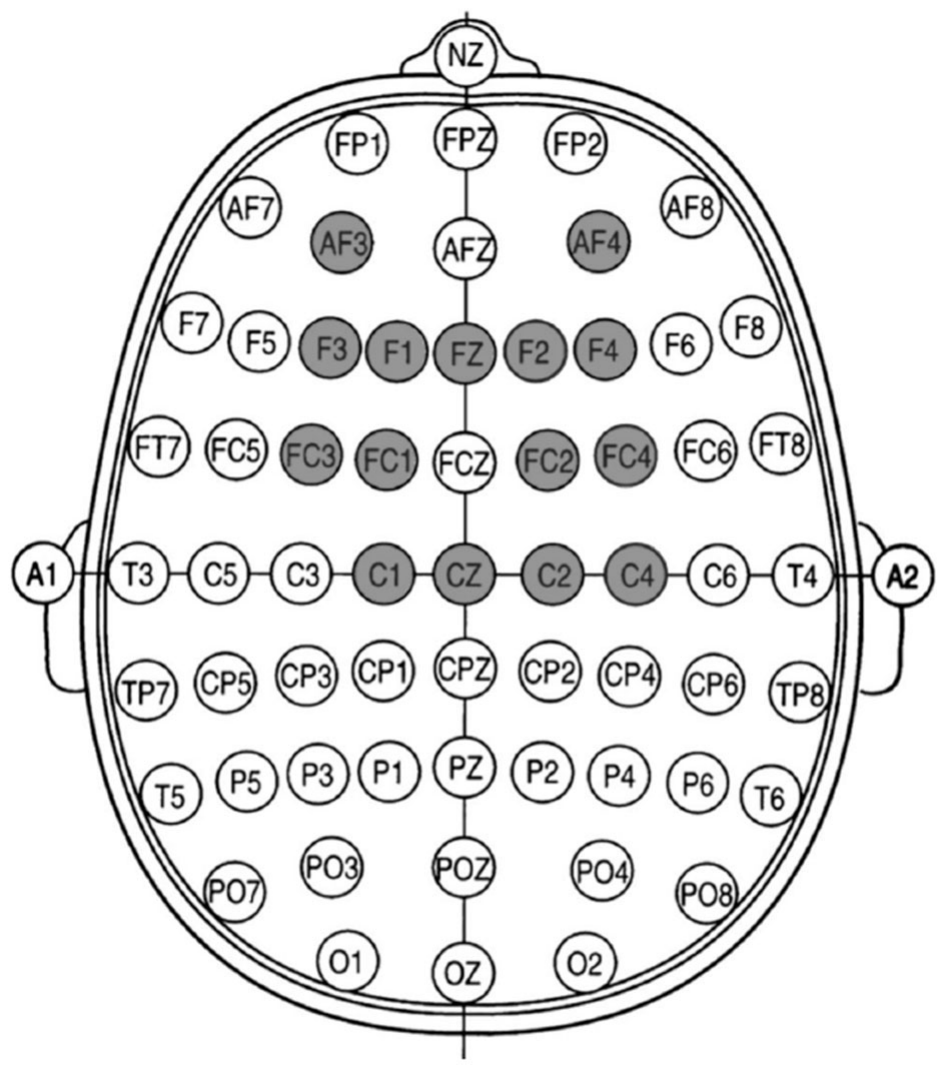
Recording channels placed according to the 10-10 system extension of the 10–20 system (Jasper, 1958).

EEG data from four ALS patients transitioning from LIS to CLIS during rest and while accomplishing an auditory paradigm (presented in the following section) were analyzed to test the algorithm, namely Patients P11, P13, P15 and P16. In addition, at least four electrodes were used to record vertical and horizontal eye movements. Figure 1 shows the location of all recording channels used for all patients. The data was acquired at a sampling rate of 500 Hz during the years 2018 and 2019.

Patient P11, a 33-year-old male with non-bulbar ALS, was diagnosed in August 2015. He last used assistive and augmentative communication (AAC) technologies in August 2017, with 10 visits recorded over 13 months. Patient P13 is a 58-year-old male diagnosed with bulbar ALS in January 2011. His last use of AAC was in January 2018, and 4 visits were recorded over a 12-month period. Patient P15, a 63-year-old female with lower motor neuron predominant ALS (ICD-10: G12.2), was diagnosed in February 2017. She last engaged with AAC in November 2018, with 2 visits recorded over 5 months. And Patient P16 is a 56-year-old male diagnosed with lower motor neuron ALS in December 2012. He last used AAC technologies in June 2018, with 2 visits recorded over 3 months. More details about the patients, the experimental setup and the data format can be found in Jaramillo-Gonzalez et al. (2021).

### 2.2 Experimental setup

Patients participated in four types of auditory sessions which were meant to be used for the development of an auditory communication system: training, feedback, copy spelling, and free spelling. The idea was to select letters to form words and sentences using eye movements. Training and feedback sessions included 20 auditory questions with known answers (10 “yes” and 10 “no”), such as “Berlin is the capital of Germany.” In the copy and free spelling sessions, patients were presented with groups of characters, each character being delivered auditorily. Patients indicated “yes” by moving their eyes and “no” by not moving them. EOG signal features corresponding to these eye movements were used to train a binary support vector machine (SVM) to identify “yes” and “no” responses, enabling patients to select letters and form sentences during feedback and spelling sessions (Tonin et al., 2020).

### 2.3 Description of the approach

**Figure 2:**
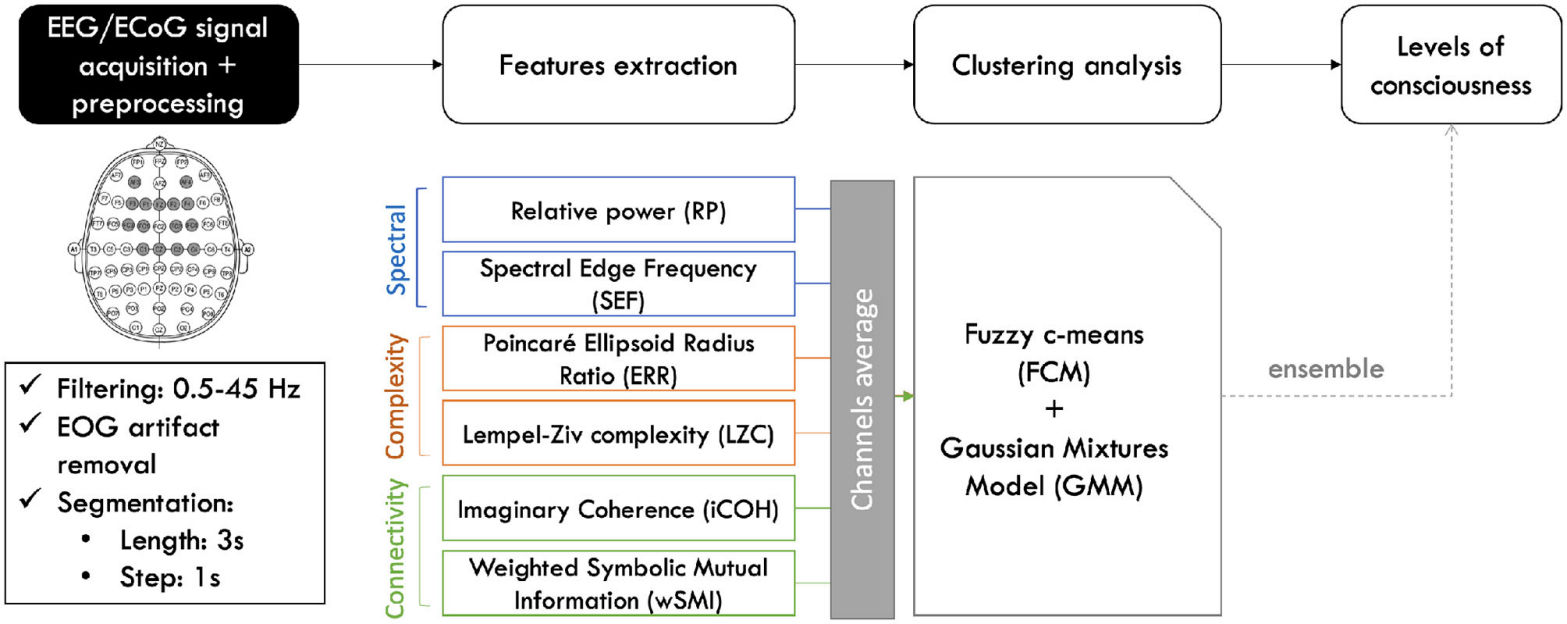
Signal processing and analysis pipeline. The recorded signals are filtered and segmented, before extracting the different features. Each feature is then averaged across selected group of channels before performing the clustering analysis. The probability that the patient is conscious is then extracted by applying a decision rule to the obtained results (Adama and Bogdan, 2023a).

The CLIS patients' NCL were determined using the approach presented in Adama and Bogdan (2023a) and illustrated in Figure 2. All analyses were performed using MATLAB. First, EOG artifacts were reduced before extracting the features of interest from the EEG signals. Then, clustering analyses were performed on these features. The results obtained from this were then used to infer the consciousness levels of the patients.

#### 2.3.1 EOG artifact removal

LIS patients still have residual eye movements that can be recorded using EOG. To reduce these artifacts on the EEG, Canonical Correlation Analysis (CCA) was used (Jiang et al., 2019). CCA is a blind source separation (BSS) technique that employs second order statistics to separate components from uncorrelated sources. To do so, it finds the linear relationship between two multi-dimensional variables and maximizes pairwise correlations across datasets. It removes muscle or ocular artifacts by investigating differences in autocorrelation between brain and muscle or EOG signals. One of its advantages is its low computational cost. The EOG artifacts were removed using MATLAB's built-in function canoncorr taking as input the raw EEG and EOG signals.

#### 2.3.2 Feature extraction

Different sets of features were computed to maximize the probability of correctly determining the patients' state as there are no ground-truth at the moment. Each of them was calculated for each EEG segment of 3-seconds length. Prior to any analysis, the signal was downsampled to 200 Hz to reduce computational costs, and filtered between 0 and 45 Hz with a third order Butterworth filter. The features comprise spectral, complexity and connectivity measures, calculated for each channel or pair of channels.

##### 2.3.2.1 Frequency-based features

The spectral features consisted of the relative powers of the θ (4–8 Hz) and β (12–30 Hz) frequency bands, and the spectral edge frequency 95% (SEF95).

The relative powers are calculated using Equation 1 (Bear et al., 2016; Wang et al., 2015).


(1) $$\begin{array}{l} RP=\frac{\overset{f_{2}}{\underset{f=f_{1}}{\sum }}S_{x}\left(f\right)}{\overset{f_{h}}{\underset{f=f_{l}}{\sum }}S_{x}\left(f\right)} \end{array}$$

where:

- *f*_1_ (resp. *f*_2_) is the lower (resp. upper) limits of the frequency band of interest,
- *f*_*l*_ = 0 Hz and *f*_*h*_ = 45 Hz,
- *S*_*x*_(*f*) is the power spectral density (PSD) of the signal *x*(*t*) at the frequency *f* (Stoica and Moses, 2005). The PSD was computed using the Welch method (Welch, 1967), implemented via the MATLAB function pwelch.

Previous research showed that there is an increase of beta power during verbal and spatial memory tasks, and has proven to be one of the best features to classify patients with disorders of consciousness (Borjigin et al., 2013). On the other hand, a global increase in both powers along with their coherence were observed during recovery of consciousness after anesthesia (Pal et al., 2015).

SEF95 is defined as the frequency below which 95% of the signal power is contained (Imtiaz and Rodriguez-Villegas, 2014; Abootalebi et al., 2009) and is calculated using Equation 2, in which *f* is the frequency and *Fs* the sampling frequency. The obtained results were then normalized by dividing them to the upper limit of the critical frequency during filtering (45 Hz).


(2) $$\begin{array}{l} \overset{SEF_{95}}{\underset{f=0}{\sum }}S_{x}\left(f\right)=0.95\overset{Fs/2}{\underset{f=0}{\sum }}S_{x}\left(f\right) \end{array}$$

This spectral feature is commonly used to determine the depth of anesthesia in healthy subjects. Deeper levels are characterized by lower SEF values (Rampil et al., 1980). For example, SEF95 above 15 Hz are representative of light anesthesia, while values between 8 and 13 Hz indicate moderate level of anesthesia. Frequencies lower than 7 Hz imply deep level of anesthesia (Touchard et al., 2019).

##### 2.3.2.2 Complexity-based features

The complexity of the EEG signals were evaluated using the Ellipsoid Radius Ratio (ERR) (Eagleman et al., 2018) and the Lempel Ziv complexity (LZC) (Lempel and Ziv, 1976). A large complexity is an indicator of an activated cortex. Consequently, an increase in the complexity of the signal indicates a higher level of consciousness, and inversely (Górska et al., 2021; Gosseries et al., 2014).

A Poincaré plot is a map where each point (*x*_*n*_, *x*_*n*+τ_) represents the value of a time series *x* at time *n* plotted against its value at time *n*+τ. In the ERR (ratio SD1/SD2) of the Poincaré plots (Eagleman et al., 2018), SD2 (resp. SD1) represents the standard deviation (SD) along the line of identity (resp. the line perpendicular to the line of identity), and describes the long term (resp. short time) variability of the signal. Both standard deviations were calculated using Equations 3, 4 (Golińska, 2013; Hayashi et al., 2014; Satti et al., 2019).


(3) $$\begin{array}{l} SD1=\frac{\sqrt{2}}{2}SD\left(x_{n}-x_{n+\tau }\right) \end{array}$$

(4) $$\begin{array}{l} SD2=\sqrt{2SD{\left(x_{n}\right)}^{2}-\frac{1}{2}SD{\left(x_{n}-x_{n+\tau }\right)}^{2}} \end{array}$$

In practice, the values of SD1 and SD2 were computed using the extended Poincaré plot algorithm developed in Satti et al. (2019) with a value of τ set to 1. The randomness of EEG signal is reduced during deep anesthesia. It is marked by a decrease of SD1, and by extension ERR (Hayashi et al., 2014). A more round shape of the ellipsoid (ERR ≈ 1) corresponds to randomness, which represent more complex signals.

On the other hand, LZC assess repetitiveness in binary sequences (Lempel and Ziv, 1976). A normalized version of LZC has been used recently to compare the consciousness levels of different types of patients with that of healthy subjects (Lee et al., 2015). To compute it, it is necessary to transform the data into a binary sequence. This was done practically by first extracting the analytic signal using the MATLAB function hilbert, and then obtaining the binary sequence by taking the mean of the absolute value of the Hilbert transform of the signal as a threshold (Schartner et al., 2015).

Let *S* = *s*_1_*s*_2_…*s*_*n*_ be the obtained binary vector of length *n*. The LZC is the number of distinct patterns in *S* as it is streamed from the left to the right (Schartner et al., 2015; Aboy et al., 2006). This was essentially done using the MATLAB toolbox calc_lz_complexity (Burns and Ramesh, 2015).

##### 2.3.2.3 Connectivity-based features

Linear and non-linear relationships between the pairs of channels were determined using the imaginary part of the coherency (iCOH) and the weighted symbolic mutual information (wSMI) in the θ bands. On one hand, the θ band plays an important part in working memory (Borjigin et al., 2013). Moreover, a decrease in coherence is observed in healthy subjects during periods of unresponsiveness induced by anesthesia (Pullon et al., 2020). On the other hand, the long-range connectivity patterns that are in theory connected to consciousness are most robustly and accurately assessed by the wSMI in the θ band (Engemann et al., 2018). Higher connectivity values represent high levels of consciousness and inversely (Bourdillon et al., 2020).

The linear relationships were estimated using the coherency, which is based on the Fourier analysis of time series signals (Kayser et al., 2009; Sakellariou et al., 2016; Nolte et al., 2004; Blinowska and Zygierewicz, 2011). Only its imaginary part is used in order to avoid volume conduction in the brain (Nolte et al., 2004). The coherency between a pair of signals *x* and *y* at frequency *f* is computed using Equation 5, where *S*_*xy*_(*f*) is the cross power spectral density of the signals, and *S*_*xx*_(*f*) and *S*_*yy*_(*f*) are the auto power spectral density of *x* and *y* respectively (Priestley, 1981).


(5) $$\begin{array}{l} C_{xy}\left(f\right)=\frac{S_{xy}\left(f\right)}{\sqrt{S_{xx}\left(f\right)\cdot S_{yy}\left(f\right)}} \end{array}$$

The non-linear relationships were assessed using wSMI, which converts the signals *x* and *y* into sequences of discrete symbols $\left(\hat{x},ŷ\right)$. This results in a decrease of the sensitivity to measurement and an increase of the efficiency of numerical computations (King et al., 2013). The symbols are determined based on the amplitude trends of *k* = 3 consecutive time points separated by a temporal separation of elements τ, leading to a total of 3! = 6 different potential symbols (*a, b, c, d, e, f*) (Lee et al., 2015). The value of τ is determined depending on the frequency of interest according to:


(6) $$\begin{array}{l} f_{max}=\frac{f_{s}}{k.\tau } \end{array}$$

where

- *f*_*max*_: maximum resolved frequency, and
- *f*_*s*_: sampling frequency (Imperatori et al., 2019; King et al., 2013).

The parameter τ is adjusted to tune wSMI sensitivity to different frequency ranges: smaller values increase sensitivity to higher frequencies, while larger values enhance sensitivity to lower frequencies. For example, setting τ = 32*ms* allows the analysis to capture slower, low-frequency patterns (King et al., 2013).

The wSMI between two signals *x* and *y* is then obtained using Equation 7.


(7) $$\begin{array}{l} wSMI\left(x,y\right)=\frac{1}{\log\left(k!\right)}\underset{\hat{x}\in \hat{X}}{\sum }\underset{\hat{y}\in \hat{Y}}{\sum }w\left(\hat{x},\hat{y}\right)p\left(\hat{x},\hat{y}\right)\log\left(\frac{p\left(\hat{x},\hat{y}\right)}{p\left(\hat{x}\right)p\left(\hat{y}\right)}\right) \end{array}$$

where $p\left(\hat{x},ŷ\right)$ is the joint probability of co-occurrence of symbols $\hat{x}$ and ŷ, $p\left(\hat{x}\right)$ and *p*(ŷ) are the probabilities of those symbols in each respective signal.

#### 2.3.3 Soft-clustering and consciousness level assessment

These features were computed for each channel or pair of channels. The results were subsequently averaged over them as illustrated in Figure 2. Only the lower part of their respective connectivity matrices without the diagonals were averaged for iCOH and wSMI measures. This resulted into a feature vector of size *n*×*p*, where *n* represents the features data samples and *p* = 7 is the dimension of the feature vector. Afterwards, the feature vector was input into two soft-clustering approaches, separately, namely Fuzzy c-mean clustering (FCM) Bezdek (1981) and Gaussian Mixture Model (GMM) (McLachlan and Peel, 2000; Ferraro and Giordani, 2020). In a regular hard clustering analysis, each data point is assigned to a specific cluster. In a soft clustering analysis, each data point can belong to different clusters with a specific membership degree. A membership degree value of 1 to a cluster means that the data point represents that cluster perfectly, while a value of 0 signifies that it is not at all representative of the cluster (Chiu, 1994; Peters et al., 2013). The sum of the membership values for all clusters is 1 (Peters et al., 2013).

FCM, a distance-based method, typically assumes spherical clusters with equal variance, GMMs, which are model-based, accommodate elliptical clusters with potentially anisotropic distributions. Using both methods at the same time enhances the robustness of clustering outcomes by mitigating bias introduced by the specific assumptions of a single algorithm. In addition, combining the results through an ensemble approach allows for the construction of consensus clusters, thereby increasing the overall reliability of the analysis.

##### 2.3.3.1 FCM

The FCM technique was first introduced in Bezdek (1981) in order to improve earlier clustering methods. The goal is to minimize an objective function:


(8) $$\begin{array}{l} J_{m}=\overset{D}{\underset{i=1}{\sum }}\overset{N}{\underset{j=1}{\sum }}\mu_{ij}^{m}||x_{i}-c_{j}|{|}^{2} \end{array}$$

where:

- *D*: number of data points,
- *N*: number of clusters,
- *m*, (*m*>1): fuzzy partition matrix that defines the cluster's fuzziness,
- *m* = 1 corresponds to a hard-clustering analysis, and
- *c*_*j*_: center of the *j*-th cluster.

The cluster centers are calculated using Equation 9.


(9) $$\begin{array}{l} c_{ij}=\frac{\overset{D}{\underset{i=1}{\sum }}\mu_{ij}^{m}x_{i}}{\overset{D}{\underset{i=1}{\sum }}\mu_{ij}^{m}} \end{array}$$

where:

- *x*_*i*_: *i*^*th*^ data point,
- μ_*ij*_: degree of membership of *x*_*i*_ in the *j*^*th*^ cluster,

(10) $$\begin{array}{l} \mu_{ij}=\frac{1}{\overset{N}{\underset{k=1}{\sum }}{\left(\frac{\left||x_{i}-c_{j}|\right|}{\left||x_{i}-c_{k}|\right|}\right)}^{\frac{2}{m-1}}} \end{array}$$

The MATLAB function fcm with the following parameters was applied to the data to perform the FCM analysis: *N* = 2 clusters (“conscious” and “unconscious”), *m* = 2 as recommended by previous research (Peters et al., 2013), the maximum number of iterations was fixed at 1000, and the minimum improvement in objective function between two consecutive iterations ϵ at 1*e*^−5^.

##### 2.3.3.2 GMM

GMM uses a Gaussian mixture distribution as a model. It assumes that the data is generated by a random statistical model that the clustering method attempts to recover (Ferraro and Giordani, 2020). Given $\text{x}=\left({\text{x}}_{1},\;{\text{x}}_{2},...,\;{\text{x}}_{\text{n}}\right)\in {\mathbb{R}}^{p}$, the random vector x_*i*_ is assumed to arise from a finite mixture of probability density functions:


(11) $$\begin{array}{l} f\left({\text{x}}_{i},\Theta \right)=\overset{K}{\underset{g=1}{\sum }}\pi_{g}\Phi \left({\text{x}}_{i}|\mu_{g},\Sigma_{g}\right) \end{array}$$

where:

- *K*: number of components (clusters);
- π_*g*_>0, (*g* = 1, …, *K*) and $\overset{K}{\underset{g=1}{\sum }}\pi_{g}=1$: mixing proportions;
- Φ = (π_1_, …π_*g*−1_, μ_1_, …μ_*g*_, Σ_1_, …, Σ_*g*_): parameter vector;
- Φ(x_*i*_|μ_*g*_, Σ_*g*_): underlying component-specific density function with parameters μ_*g*_; σ_*g*_, *g* = 1, …, *K*.

A cluster is represented by a specific mixture component density that is associated to a specific parametric class. The method uses maximum likelihood optimisation to estimate the parameters in Φ (Ferraro and Giordani, 2020). Ellipsoidal clusters centered at the mean vector μ_*g*_ are generated by the model represented in Equation 11. The other geometrical properties of each cluster are controlled by the parameter σ_*g*_. Difference of means in the different component models suggest that the model distinguishes among the *K* classes (McLachlan and Peel, 2000).

The MATLAB function fitgmdist was used to fit GMMs to the data. The same parameters as for the FCM were used. Furthermore, the MATLAB function posterior was applied to estimate the component-membership posterior probabilities (Stahl and Sallis, 2012).

##### 2.3.3.3 Normalized consciousness level (NCL) determination

Each of these two clustering methods produced degrees of membership to each of the states of interest. In this study, the consciousness level is defined as the degree of membership of each data sample *i* to the cluster representing “conscious” states (Adama and Bogdan, 2023a,b). The premise is that higher consciousness levels are characterized by:

- an increase of power in the θ (*RP*_θ_) and β bands (*RP*_β_),
- a value of SEF95 superior to the α band,
- a higher ERR value of the Poincaré plots,
- a larger LZC value,
- A greater value of iCOH and wSMI in the θ band.

The resulting degrees of membership obtained from the two clustering approaches were subsequently combined using a product ensemble (Equation 12, in which *P*(*c, m*_1_) (resp. *P*(*c, m*_2_)) represents the probability that the data point *i* belongs to cluster *c* in partition *m*_1_ (resp. *m*_2_)).


(12) $$\begin{array}{l} P_{prod}\left(c,m_{1}m_{2}\right)=prod\left(P\left(c,m_{1}\right),P\left(c,m_{2}\right)\right) \end{array}$$

The NCL values are the elements in *P* from Equation 12 that represent the membership value to the “conscious” cluster.


(13) $$\begin{array}{l} NCL=P_{prod}\left(c=”conscious”\right) \end{array}$$

It is important to clarify that the approach used here intends to introduce an alternative perspective on the assessment of consciousness levels focused primarily on (completely) locked-in patients.

### 2.4 Evaluation of the approach

The clustering results were evaluated by assessing the clusters separability (how well separated they are). Moreover, the approach itself was evaluated by comparing the obtained results to the performance accuracy achieved by the patient during the experiments. It is expected that high accuracies will be obtained during states with higher levels of consciousness. However, the opposite is not true in this case as the experiment is based on EOG, and the ability to perform voluntary muscle movements are reduced as the condition progresses, although the cognitive functions remain almost intact (Bear et al., 2016; Posner et al., 2007).

#### 2.4.1 Clustering analysis validation

To validate the effectiveness of the clustering methods, partition coefficient and partition entropy metrics were employed. These validation measures were used to ensure that the soft clustering models effectively differentiate between the conscious and unconscious states.

##### 2.4.1.1 Partition coefficient (PC)

Partition coefficient (PC) assesses the clarity of cluster assignments. It computes the relative average of fuzzy intersection between pairs of fuzzy subsets in *U* by their algebraic product. PC is a maximization index which value ranges between 1/*c* and 1. Lower values suggest a very fuzzy clustering while a value equal to 1 represents a well-defined clustering (Bezdek, 1973, 1974; Eustquio and Nogueira, 2020).


(14) $$\begin{array}{l} PC\left(U\right)=\frac{1}{n}\overset{c}{\underset{i=1}{\sum }}\overset{n}{\underset{k=1}{\sum }}{\left(A_{i}\left(x_{k}\right)\right)}^{2} \end{array}$$

where: *U*: fuzzy pseudo-partition defined as *U* = [*A*_*i*_(*x*_*k*_)]. *A*_*i*_(*x*_*k*_): membership degree of *x*_*k*_ in cluster *A*_*i*_, 1 ≤ *i* ≤ *c*. *c*: number of clusters, *n*: number of observations to be clustered, *x*_*k*_: an observation to be clustered, 1 ≤ *k* ≤ *n*. In this study, *c* = 2 (conscious vs. unconscious). Consequently, PC values range between 0.5 and 1.

##### 2.4.1.2 Partition entropy (PE)

Partition entropy (PE) provides insight into the fuzziness of the clusters, indicating the degree of overlap between them. It is a minimization index that uses Shannon's entropy function to describe the fuzzy uncertainty in each object *x*_*k*_. Its values range from 0 to 1, in which a value of 0 represent complete well-defined and non-overlapping clusters, and 1 displays maximum uncertainty between them (Bezdek, 1975; Eustquio and Nogueira, 2020). The fuzzy uncertainty in a pseudo-partition *U* is measured using the following equation:


(15) $$\begin{array}{l} PE\left(U\right)=-\frac{1}{n}\overset{c}{\underset{i=1}{\sum }}\overset{n}{\underset{k=1}{\sum }}A_{i}\left(x_{k}\right)log\left(A_{i}\left(x_{k}\right)\right) \end{array}$$

#### 2.4.2 Proxy conscious states based on known feature values

The performance of NCL was evaluated by comparing threshold-based proxy values derived from the literature, which characterize features typically associated with conscious states. These values were interpreted as lower-bound indicators of consciousness and used to construct a reference matrix. This matrix had the same number of observations as the original feature matrix and was generated by applying the defined threshold to each corresponding feature. Specifically, for each feature, if the observed value exceeded the respective threshold, the corresponding entry in the proxy matrix was assigned a value of one, indicating that the feature supports the presence of consciousness in that observation. Otherwise, a value of zero was assigned. Subsequently, for each observation, the average across all proxy features was computed, yielding a continuous index that reflects the degree of support for consciousness based on the known thresholds. This index was then compared to the NCL output.

The threshold values were selected based on published findings. For instance, RPtheta values in patients diagnosed with an MCS were reported as follows in Lehembre et al. (2012): 0.0523 ± 0.02 in the frontal region, 0.0648 ± 0.03 in the posterior region, 0.0589 ± 0.03 in the left hemisphere, and 0.058 ± 0.02 in the right hemisphere. SEF95 values exceeding 8 Hz, typically in the alpha band or above, have been associated with conscious brain activity (Touchard et al., 2019). LZC values around 0.34 ± 0.03 have been reported in healthy controls during eyes-closed resting states (yu Wu et al., 2011). Theta-band iCOH values for MCS patients were found to be 0.118 ± 0.02 for inter-hemispheric connections, 0.134 ± 0.02 for frontal-to-posterior connections, 0.12 ± 0.03 in the left hemisphere, and 0.114 ± 0.02 in the right hemisphere (Lehembre et al., 2012). wSMI values in healthy subjects have been reported to average 0.088 (King et al., 2013).

No reference values were identified in the literature for RPbeta or Poincaré ERR; these features were therefore excluded from the threshold-based proxy computation.

Based on the evidence, the following values were used in this study as minimal thresholds for features indicative of consciousness: RPtheta = 0.07, SEF95 = 8 Hz, LZC = 0.4, iCOH = 0.14, and wSMI = 0.088. These thresholds were applied to construct the binary proxy matrix, from which the average score per observation was derived and subsequently compared against the NCL output to evaluate its agreement with established neurophysiological markers of consciousness.

#### 2.4.3 Statistical analysis

##### 2.4.3.1 Correlation between proxy conscious states and NCL

For each patient, the association between the proxy-based consciousness scores and the NCL output was assessed using Spearman's rank correlation coefficient. This method was selected due to its ability to assess monotonic relationships without assuming a linear relationship or normal distribution of the data, and was implemented using MATLAB's corr function.

##### 2.4.3.2 Correlation between estimated NCL and performance accuracy

The clustering results determining the patients' NCLs were compared with their performance when executing the EOG-based communication task described in Section 2. The working hypothesis being that the former is positively correlated with the latter; in other words, high prediction accuracies are obtained when the patient is conscious. However, since the inability to perform voluntary eye movements do not translate to patients being unconscious, the inverse is not true. Spearman's rank correlation coefficient was also used to do so (Spearman, 1904).

## 3 Results

The goal of this research is to assess LIS patients' consciousness states using an NCL measure. As there are no ground truth, different features were computed from the patients' EEG recordings in order to maximize the probability of correctly determining their states. The normalized consciousness level is obtained by performing a soft clustering analysis on the computed features averaged across all channels and pairs of channels. As we are interested in conscious and unconscious states, the number of clusters for both clustering approaches was set to two. This choice is also supported by the Calinski-Harabasz index (CHI) (Calinski and Harabasz, 1974), computed for each recording session separately for each patient. This indicated two as the optimal number of clusters in most cases for both algorithms (see Supplementary material). The results obtained from each patient is presented in this section.

### 3.1 Normalized consciousness levels

#### 3.1.1 Patient P11

**Figure 3:**
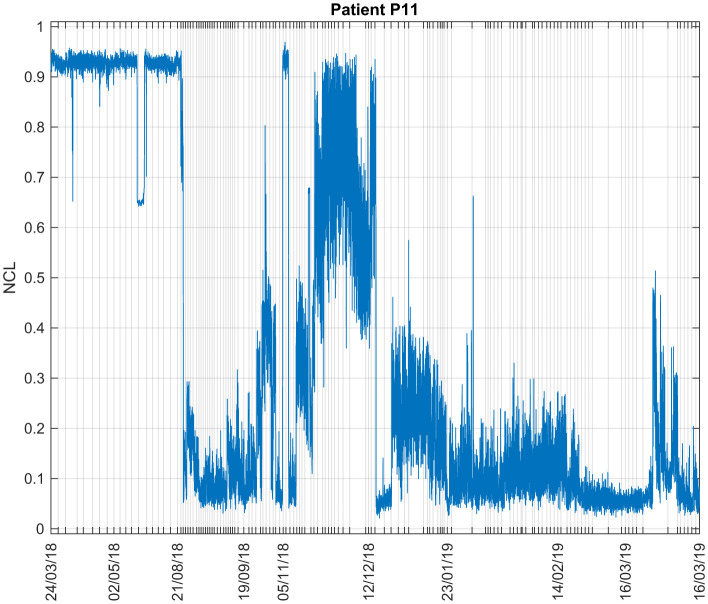
Consciousness levels estimations during all sessions for Patient P11. The ticks in the x-axis represent the start of each recording session.

Figure 3 shows the NCL estimations for Patient P11 during all his recording sessions. The data of this patients consisted of EEG and EOG recordings from March 2018 to March 2019, consisting respectively of 6 and 4 different days of experiments. The predicted consciousness levels were very variable from session to session. During March and May 2018, high NCL values were observed, averaging to 0.9070. Lower values were then obtained for August and September, with an average value of 0.2136, but in November, the NCL average value reached 0.6490 then decreased again in December (average: 0.1152). As can be observed on the figure, NCL values were continuously low from January 2019 on. A decrease in consciousness level as time progresses could suggest cognitive decline.

#### 3.1.2 Patient P13

**Figure 4:**
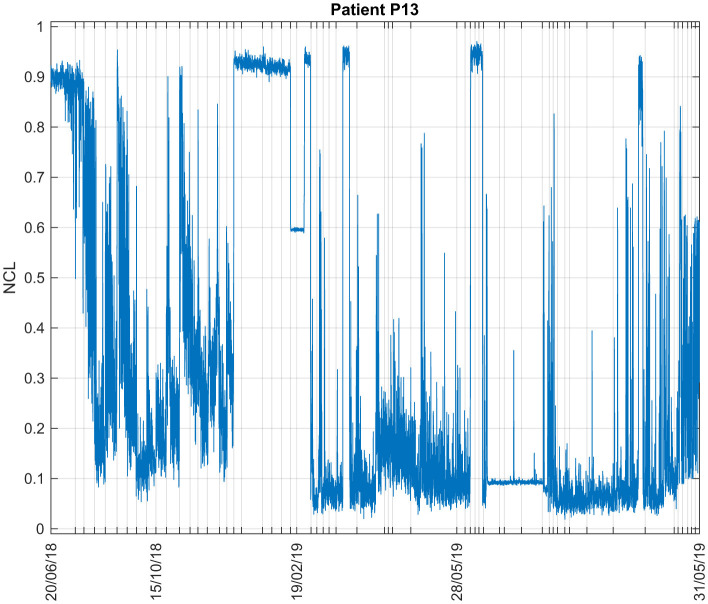
Consciousness levels estimations during all sessions for Patient P13. The ticks in the x-axis represent the start of each recording session. The shown dates are the starting dates of each visit, each visit comprises several days.

The data of Patient P13 were recorded in 2018 (2 days) and 2019 (3 days). Figure 4 shows his NCL during all days and sessions of recording. The levels are very variable from session to session. High values were observed at first, but then consistently decrease afterwards. More precisely, the average NCL value for this patient in 2018 was 0.5549, and decreased to an average of 0.3663 in 2019.

#### 3.1.3 Patient P15

**Figure 5:**
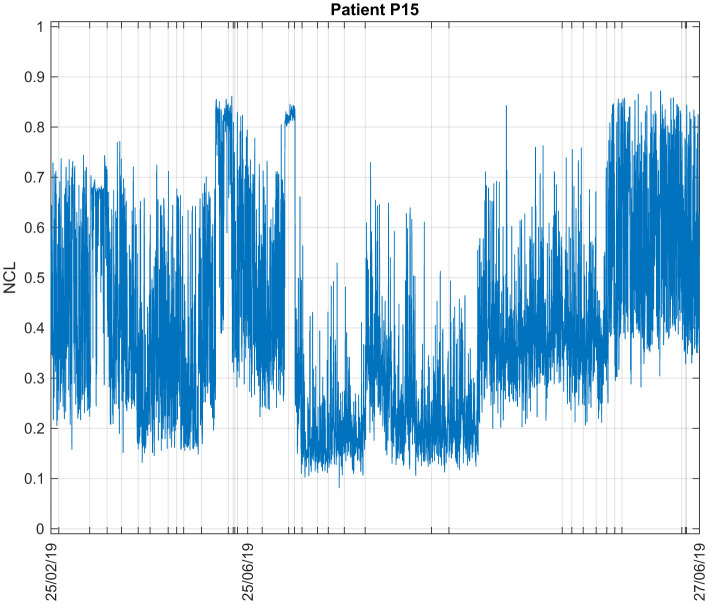
Consciousness levels estimations during all sessions for Patient P15. The ticks in the x-axis represent the start of each recording session.

The data available for this patient consist of 4 days in 2019. The obtained NCL are shown in Figure 5. Overall, its average value during all sessions is 0.4703 and the values do not vary much. This suggests that the patient was relatively in the same state (conscious or unconscious) throughout the experiments.

#### 3.1.4 Patient P16

**Figure 6:**
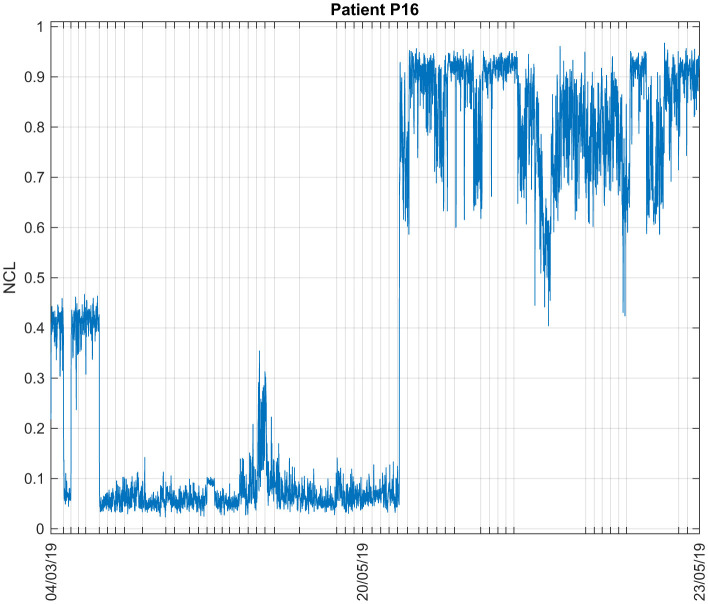
Consciousness levels estimations during all sessions for Patient P16. The ticks in the x-axis represent the start of each recording session.

A total of 3 days recordings from 2019 were available for Patient P16. Figure 6 illustrates the NCL during all sessions. There are three different patterns observed: at first, the NCL average value is 0.3335 in March 2019, then its values decreased to values lower than 0.1. During the last days of experiments, an increase was observed with an average value of 0.8158. This suggests that the patient was only conscious during the last days of experiments.

### 3.2 Correlation between NCL and features

**Figure 7:**
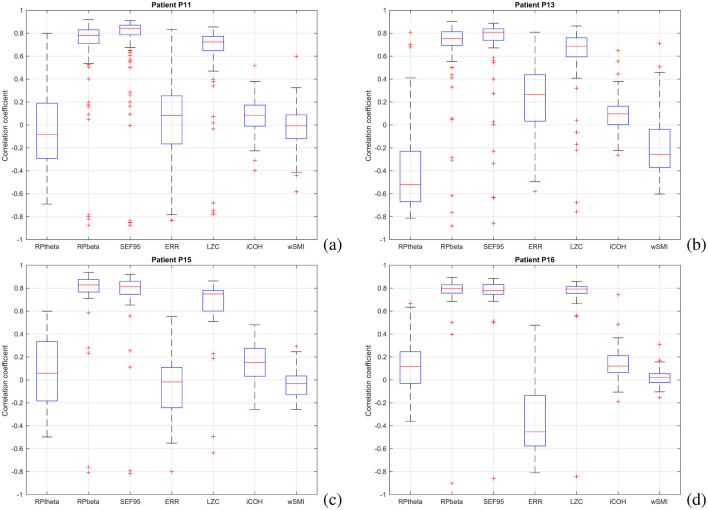
Correlation between features and estimated consciousness levels for all patients. **(a)** Patient P11, **(b)** Patient P13, **(c)** Patient P15, **(d)** Patient P16.

Correlation coefficients between the features and the NCL were computed for each session, and the results are illustrated in Figure 7.

For Patient P11, frequency-based features (*RP*_β_ and SEF95) and LZC complexity contribute most positively to the final results, while connectivity features, particularly wSMI, have the least impact. SEF95 (median: 0.8431) was the highest contributor, whereas wSMI (-0.0075) had the lowest influence. For Patient P13, Figure 7b shows the correlation coefficients between features and NCL. As with Patient P11, *RP*_β_, SEF95, and LZC exhibit the strongest correlations. *RP*_θ_ also plays a significant but negative role, aligning with existing literature, as theta rhythms typically emerge during deep sleep when cortical neurons are not involved in information processing (Niedermeyer, 2005). Similarly, for Patient P13, SEF95 (median: 0.8092) had the highest contribution, while wSMI (0.0964) had the lowest. For Patient P15, Figure 7c presents the correlation coefficients between features and NCL across all recording sessions, confirming the trends observed in Patients P11 and P13. Frequency-based features (*RP*_β_ and SEF95) and the complexity feature LZC show the strongest positive correlations, while wSMI contributes minimally. For Patients P15 and P16, *RP*_β_ had the highest impact (median: 0.8281 and 0.7947, respectively), whereas wSMI had the least influence (−0.0178 and 0.0233, respectively).

To summarize, frequency-related features (*RP*_β_ and SEF95) and complexity (LZC) appear to be the most significant contributors for all patients. A reduction in β power suggests a shift toward a resting-state brain, whereas an increase indicates heightened brain engagement in information processing (Gazzaniga et al., 2018; Niedermeyer, 2005). Furthermore, SEF95 values below 7 Hz are typically associated with deep anesthesia, signifying low-consciousness states (Touchard et al., 2019). Additionally, lower complexity values reflect less random and therefore less intricate brain signals. Higher levels of consciousness are associated with increased signal complexity, while lower complexity corresponds to reduced conscious states (Górska et al., 2021).

On the other hand, the less contributing factors appear to be the connectivity features. Specifically, for Patient P11, the wSMI feature has a median correlation value of −0.075. Similarly, for Patient P13, the iCOH feature shows a median correlation value of 0.0964. In the case of Patient P15, the ERR feature has a median correlation value of −0.0178. Lastly, for Patient P16, the wSMI feature presents a median correlation value of 0.0233. During wakefulness, efficient information processing is associated with increased connectivity values. However, alterations in connectivity values may lead to disrupted functional connectivity patterns, which could contribute to impaired sensory processing in states of reduced consciousness (Desjardins et al., 2017). This suggests that the previous observation that connectivity features, such as wSMI and iCOH, appear to be less contributing factors in specific patients, may indicate a potential link between altered connectivity and reduced cognitive function.

### 3.3 Correlation between NCL and proxy conscious states

Given the absence of an objective ground-truth for consciousness in patients with LIS and CLIS, the performance of the proposed consciousness assessment method was initially evaluated using proxy-based consciousness scores. This evaluation involved computing Spearmans rank correlation coefficient between the NCL values and the proxy scores for each individual patient.

For Patient P11, the analysis revealed a Spearman correlation coefficient of ρ = −0.0517, with a highly significant *p*-value of 6.43 × 10^−55^. Although this result is statistically significant, the very low magnitude of the correlation suggests that there is no meaningful relationship between the NCL and the proxy-based conscious state score in this case. The statistical significance is likely a consequence of the large number of data points rather than a reflection of an actual association.

In contrast, Patient P13 showed a strong positive correlation between the NCL and the proxy scores, with a Spearman coefficient of ρ = 0.7417 and a *p*-value of zero. This result indicates a clear and statistically robust monotonic relationship, suggesting that increases in the NCL are reliably associated with increases in the proxy-based estimate of consciousness for this patient.

A similar pattern was observed for Patient P15, where the Spearman correlation coefficient was ρ = 0.7661, also with a *p*-value of zero. This strong positive correlation again supports the validity of NCL as a marker of consciousness, at least in this individual case.

For Patient P16, the correlation coefficient was ρ = 0.4971, with a *p*-value of zero. Although weaker than in Patients P13 and P15, this moderate positive correlation still indicates a meaningful relationship between the two measures, suggesting partial alignment between NCL and the proxy consciousness index.

Taken together, these results demonstrate that the NCL correlates strongly with proxy-based markers of consciousness in several cases, particularly for Patients P13 and P15. However, the absence of a meaningful correlation in Patient P11 highlights the presence of inter-individual variability. Such variability may arise from differences in clinical condition, signal quality, or how well the proxy thresholds reflect consciousness in specific patients. These findings suggest that while NCL appears to capture relevant aspects of conscious processing in many cases, additional patient-specific factors may influence its effectiveness and should be further explored in future analyses.

### 3.4 Correlation between NCL and prediction accuracy

Additionally, the performance of the proposed consciousness assessment method was also evaluated using the prediction accuracy obtained while the patients were performing the EOG-based communication tasks described in Section 2. The assumption is that higher accuracy reflects a conscious state. However, low accuracy does not necessarily mean the patient is unconscious, especially considering the progression from LIS to CLIS. Data on classification and prediction accuracy for the EOG-based communication system can be found in the original publication (Tonin et al., 2020; Jaramillo-Gonzalez et al., 2021).

Table 1 shows the correlation coefficients values between NCL and prediction accuracy. The prediction accuracy for Patient P11 ranges between 15% and 100%, with an average accuracy of 71.82%. A statistically significant correlation was observed between median NCL and prediction accuracy, with a correlation coefficient of *r* = 0.3779 and a *p*-value of 0.0053 (*p* < 0.05). For Patient P13, prediction accuracy varies between 5% and 100%, with a mean accuracy of 64.55%. The correlation between the median NCL and the prediction accuracy is *r* = 0.1211, and the *p*-value of 0.6215 indicates a lack of statistical significance. In the case of Patient P15, prediction accuracy fluctuates from 5% to 95%, with a median accuracy of 64.38%. The correlation between median NCL and prediction accuracy is negative, with *r* = −0.2130 and a *p*-value of 0.4646, showing no significant relationship. Prediction accuracy for Patient P16 spans from 0% to 100%, with a median value of 66.59%. The correlation between median consciousness levels and prediction accuracy is *r* = 0.1610, with a *p*-value of 0.4978, indicating no statistically significant correlation.

**Table 1:** Average correlation coefficient values between features and prediction accuracy during the EOG-based communication for all patients.

	**P11**	**P13**	**P15**	**P16**
*RP*_θ_	−0.4406 ^(*)^	−0.0654	−0.3341	0.1321
*RP*_β_	−0.0531	0.0292	0.2960	−0.2293
SEF95	0.0820	0.1413	0.226	−0.0437
ERR	0.0858	0.4103 ^(**)^	0.1727	0.2179
LZC	0.1927	0.5020 ^(*)^	0.1660	0.1526
iCOH	−0.2248	−0.2227	−0.0022	0.0380
wSMI	−0.2	0.1237	−0.1435	−0.1435
NCL	0.3779 ^(*)^	0.1211	−0.2130	0.1610

^(*)^ means significant with *p* < 0.05, and ^(**)^ with *p* < 0.1.

All NCL estimations are positively correlated with the prediction accuracies, except for Patient P15. For Patient P11, the correlation strength is moderate and statistically significant, while it is weak for the other patients. A moderate (or weak) increase or decrease in NCL corresponds to a similar trend in prediction accuracy. This correlation strength likely results from a decline in accuracy due to the EOG-based nature of the experiment. As patients transition to CLIS and lose eye movement capabilities, accuracy drops, even though their cognitive functions largely remain intact (Posner et al., 2007).

### 3.5 Clustering fitness

Different measures were used to assess the performance of the clustering results. The analyses were done offline, on all available data for each patient separately. Table 2 shows the values of the PE and PC for all patients. Typically, PC values range from 0.5 (in this case) to 1, while PE values range from 0 to 1. Well-defined clusters are characterized by a high PC value and a low PE value (Bezdek, 1973, 1974, 1975). For Patient P15, PC and PE results indicate poor clusters separation, as can also be seen in Figure 5 with the average values of NCL. The patient was either conscious or unconscious during all sessions. The values of the features suggest the latter as can be seen in the upper part of Table 2. Low values of the individual features correspond to lower consciousness states. For the Patients P11, P13 and P16, conscious and unconscious states were clearly separated.

**Table 2:** Median values of the different features extracted from the EEG data for all patients.

	**P11**	**P13**	**P15**	**P16**
*RP*_θ_	0.3478	0.3647	0.4269	**0.3119**
*RP*_β_	0.1662	0.1952	**0.1129**	0.1676
SEF95	0.5642	0.6293	**0.4464**	0.5859
ERR	0.8114	0.5917	**0.5081**	0.6904
LZC	0.0995	0.0834	**0.0628**	0.0877
iCOH	0.2804	0.2597	0.2380	**0.1481**
wSMI	**−0.0073**	−0.0130	−0.0075	−0.0194
PC	0.7998	0.7793	0.6312	0.7877
PE	0.3364	0.3639	0.5501	0.3499

Values in bold represent the lowest values inter-patients. Partition coefficient (PC) and partition entropy (PE) values for all patients.

## 4 Discussions and conclusion

This paper investigated the conscious states of four LIS patients using their EEG and an approach providing a normalized consciousness level value between 0 and 1 representing respectively unconscious and conscious states. This approach was first introduced in Adama and Bogdan (2023a,b) and was capable to successfully estimate the consciousness level of one CLIS patient with a correct answer rate of 88.89%. Using this approach to analyse consciousness during sleep in patients with disorders of consciousness, an accuracy of up to 70.11% was achieved for patients in a vegetative state, and over 80% for those in a minimally conscious state, relative to their eyes-open or eyes-closed conditions (Adama and Bogdan, 2023b). By using different sets of features, the idea is to maximize the probability of correctly assessing these patients' actual state considering the lack of ground truth.

To evaluate the performance of the proposed method in the absence of a definitive ground truth for consciousness, the Normalized Consciousness Level was compared against a proxy-based consciousness score derived from known physiological features, and the prediction accuracy achieved during eye movement-based communication tasks. In addition, the quality of the unsupervised clustering was assessed using clustering validity indices, specifically the Partition Coefficient and the Partition Entropy.

The correlation analysis between NCL and proxy-based consciousness scores revealed consistent and interpretable results across most patients. Strong positive correlations were observed in Patients P13 and P15, while Patient P16 demonstrated a moderate positive association. These findings suggest that the NCL measure aligns well with established neurophysiological markers that are generally considered indicative of consciousness. Notably, the lack of correlation in Patient P11, despite statistical significance, may indicate individual variability or a mismatch between the proxy thresholds and the patient's specific neurophysiological profile. Overall, the correlation between NCL and proxy scores provides preliminary evidence supporting the validity of the proposed measure in capturing consciousness-related neural dynamics, even in the absence of explicit behavioral responsiveness.

In addition to proxy comparisons, the relationship between NCL and EOG-based communication system prediction accuracy was also examined. The patients' performance during the experiments can be found in Tonin et al. (2020); Jaramillo-Gonzalez et al. (2021). For Patient P11, a significant positive correlation was observed, indicating that higher NCL values were associated with better communication performance. Patient P13 showed a positive but less pronounced correlation, while Patient P15 exhibited a weak negative correlation that did not reach statistical significance. Patient P16 showed a similarly weak but positive association. These variable results likely reflect the influence of external factors on the EOG-based system performance, particularly the reliance on eye movement signals. As patients progress toward a complete locked-in state and gradually lose the ability to generate reliable eye movements, the accuracy of such interfaces tends to decline, even if cognitive processing remains relatively intact. This dissociation may explain the reduced correspondence between NCL and accuracy in some cases.

Clustering analysis further highlighted inter-patient variability. Clear and well-separated clusters were identified for Patients P11, P13, and P16, suggesting distinct neural states that the model could differentiate effectively. In contrast, clustering results for Patient P15 were notably poor, as reflected by the corresponding partition coefficient and partition entropy values. This may indicate that the neural activity patterns for this patient were less structured or more variable, potentially due to lower signal quality or a less consistent cognitive response.

Taken together, the results suggest that the proposed model demonstrates promising performance in capturing neural markers of consciousness and differentiating states in most patients. Its ability to correlate with proxy-based indicators and, to some extent, with EOG-based system performance underscores its potential as a tool for monitoring residual consciousness in LIS and CLIS populations. However, the variability observed across individuals highlights the need for personalized calibration and further validation. The limited effectiveness of the NCL approach in certain cases likely reflects inter-subject neurophysiological variability, or a mismatch between the fixed thresholds and the patients individual neural signature. Additionally, a lack of motivation or engagement during the task may also contribute. These factors highlight the importance of a personalized and adaptive approach.

It is important to note that the dataset used in this study does not include scores from the Revised Amyotrophic Lateral Sclerosis Functional Rating Scale (ALSFRS-R), which is commonly employed to evaluate the level of functional impairment in ALS patients. However, the ALSFRS-R primarily assesses motor function and daily activities, and it does not offer a direct or precise measure of communication ability. For instance, even a patient with an ALSFRS-R score of zero may retain some capacity for communication through minimal voluntary movements, such as eye gaze control (Tonin et al., 2020). As a result, the absence of observable motor responses should not be equated with the absence of conscious awareness or the inability to communicate. Traditional behavioral assessments are similarly limited in their ability to detect covert consciousness, especially in patients who do not exhibit overt signs of awareness. Such tools may fail to capture the presence of preserved cognitive functions when voluntary motor output is severely compromised or entirely absent.

The clinical relevance of the proposed approach lies in its potential to provide individualized, objective indicators of consciousness in patients who are otherwise unable to express themselves behaviourally. By offering a patient-specific assessment of neural activity patterns consistent with conscious processing, this method may support more accurate evaluations of residual awareness. This, in turn, could assist family members and clinical staff in better understanding the patient's current cognitive state and guide decisions about attempting communication, thus contributing meaningfully to the patients quality of life in the complete locked-in state.

Patients with LIS due to ALS, such as those included in this study, are generally not expected to experience functional recovery. As the disease progresses and patients transition into CLIS, the absence of interaction with the external environment may contribute to cognitive decline. However, it has been shown that providing communication means can help delay such decline (Secco et al., 2021).

Moreover, the establishment of a reliable and functional communication system has been shown to significantly improve quality of life. Recent findings emphasize this impact, with evidence demonstrating that patients who were supported with communication technologies reported enhanced wellbeing and sustained levels of perceived quality of life (Voity et al., 2024). Notably, a longitudinal study conducted between 2007 and 2013 found that 70% of patients reported stable or improved quality of life when given the opportunity to communicate (Rousseau et al., 2015).

These observations underscore the critical importance of developing and refining BCI-based communication systems tailored for patients in advanced stages of ALS. Providing such individuals with the ability to interact with their relatives, caregivers, and medical staff contributes meaningfully to their psychological and emotional wellbeing. Furthermore, continuous monitoring of brain activity in CLIS patients may offer additional benefits, enabling adaptive and personalized interventions that enhance care and improve the patients overall quality of life.

This research represents a step toward improving the care and quality of life for CLIS patients. One of the main benefits of NCL is that it does not require the patient to actively perform a task. This helps reduce fatigue, which is a major concern in severely disabled patients. It also lowers the chance of misdiagnosing a conscious patient as unconscious, which can happen when tests rely too much on movement or speech. For all these reasons, NCL should be seen as a new and separate tool that complements existing clinical assessments by focusing on consciousness detection in patients who cannot move or speak. It is however important to note its limitations, given the limited sample size and the lack of ground-truth. Indeed, the extreme rarity of data from LIS and CLIS patients is largely due to the low prevalence of the condition. The prevalence of ALS is approximately 4.42 per 100,000 people (Xu et al., 2020). Incidence rates vary by country, ranging from 0.26 (Ecuador) to 23.46 per 100,000 person-years (Japan) (Wolfson et al., 2023). LIS following ALS is even rarer, with no known exact incidence or prevalence (Schnetzer et al., 2023). Additionally, only a small number of cases consented to take part on the experiments. Nonetheless, further investigation with more patients is needed to validate these findings.

In addition, another limitation concerns the use of clustering. Given the predefined number of cluster (two in this case), this approach will always partition the data into two clusters regardless of whether the underlying data genuinely reflect distinct states. This means that even if a patient remained in a stable state throughout a session, clustering would still impose artificial divisions. Although clustering metrics may indicate poor partition quality in such scenarios, some result will always be produced. The distributions of proxy features associated with consciousness were then evaluated to mitigate this, providing important contextual information to support the interpretation of clustering outcomes.

This study includes features previously validated in sleep and anesthesia research. Using diverse feature types aims to improve the accuracy of predicting patients' states. Hence, future works will focus on investigating other potential meaningful features based on frequency and complexity, such as gamma relative powers, fractal dimension, and entropy as frequency and complexity-based features appeared to be the most meaningful characteristics in this research, as opposed to the connectivity measures.

## Data Availability

Publicly available datasets were analyzed in this study. This data can be found here: doi: 10.5281/zenodo.3605395.
