# Supplementary material for: Assessing consciousness in patients with locked-in syndrome using their EEG
Source: Front Neurosci. 2025 Sep 11;19:1604173. doi: 10.3389/fnins.2025.1604173 (PMC12460305; doi:10.3389/fnins.2025.1604173)
Supplement: Supplementary file 1 [file Data_Sheet_1.pdf]

Supplementary material to Assessing consciousness in patients with locked-in syndrome using their EEG

OPTIMAL NUMBER OF CLUSTERS USING THE CALINSKI-HARABASZ INDEX

To evaluate the clustering structure of the feature vector, two soft clustering methods were independently applied: Fuzzy C-Means (FCM) clustering Bezdek (1981) and Gaussian Mixture Models (GMM) McLachlan and Peel (2000); Ferraro and Giordani (2020). Unlike traditional hard clustering, where each data point is assigned to a single cluster, soft clustering allows each data point to be associated with multiple clusters to varying degrees, reflecting uncertainty or overlap in the data structure. To assess the compactness and separation of the resulting clusters, the Calinski-Harabasz Index (CHI), also called variance ratio criterion (VRC), was employed Calinski and Harabasz (1974). The CHI quantifies clustering quality by comparing the between-cluster dispersion to the within-cluster dispersion, with higher values indicating better-defined and more distinct clusters.

Practically, MATLAB's `evalclusters` function was used to evaluate both methods by testing multiple values of clusters k, specifically $k = 1, \ldots, 5$. More specifically, k-means was employed in place of FCM for evaluation purposes, as it represents a hard clustering approximation of the FCM algorithm.

The CHI is calculated using the following equation:

$$VRC_k = \frac{SS_B}{SS_W} \cdot \frac{(N-k)}{(k-1)} \tag{S1}$$

where:

- SS_B: overall between-cluster variance,

$$SS_B = \sum_{i=1}^{k} n_i ||m_i - m||^2 \tag{S2}$$

  where n_i is the number of observations in cluster i, m is the mean of all observations, and m_i is the centroid of cluster i. $||.||$ is the Euclidean distance between the two vectors.
- SS_W: overall within-cluster variance,

$$SS_W = \sum_{i=1}^{k} \sum_{x \in c_i} ||x - m_i||^2 \tag{S3}$$

  where x represents a data point and c_i represents cluster i.
- k: number of clusters,
- N: number of observations.

A higher VRC indicates well-separated (with large between-cluster distances) and compact clusters (with small within-cluster distances).

Figures S1, S2, S3 and S4 respectively showed the results of the analysis for Patients P11, P13, P15 and P16 for the FCM and GMM clustering methods.

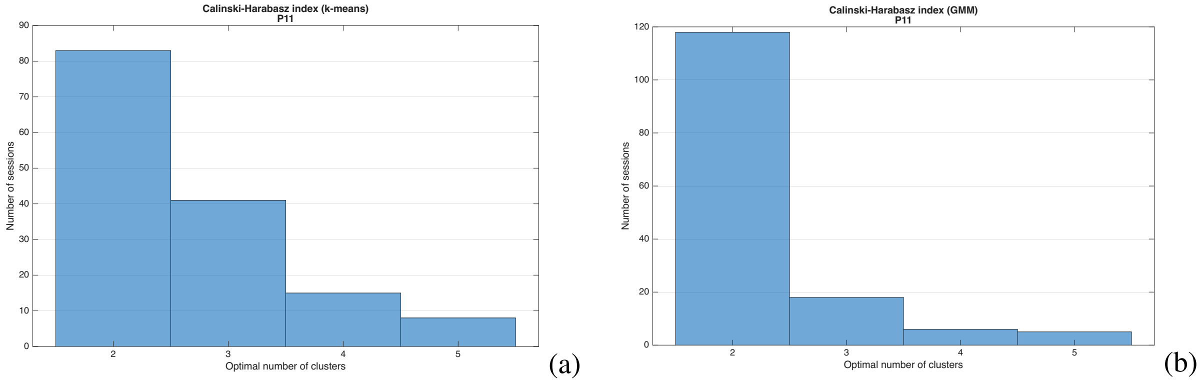

Figure S1. Optimal number of clusters for Patient P11 using **(a)** k-means, **(b)** GMM.

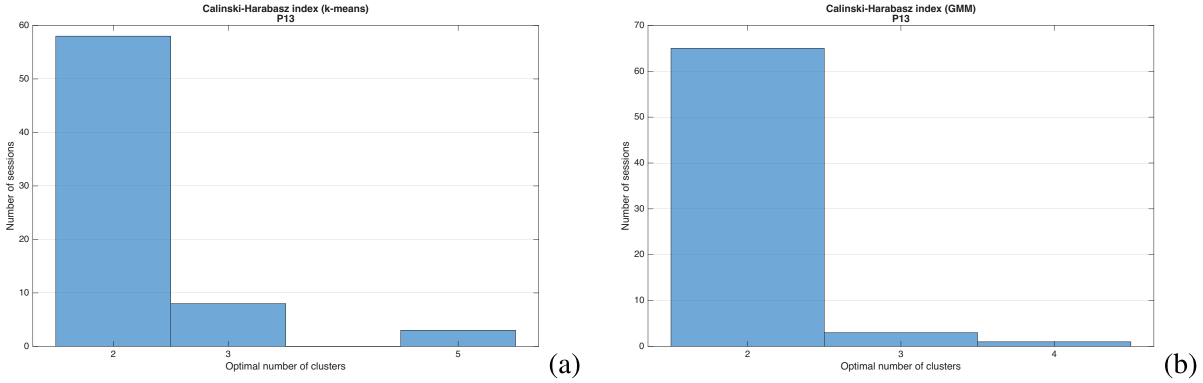

Figure S2. Optimal number of clusters for Patient P13 using **(a)** k-means, **(b)** GMM.

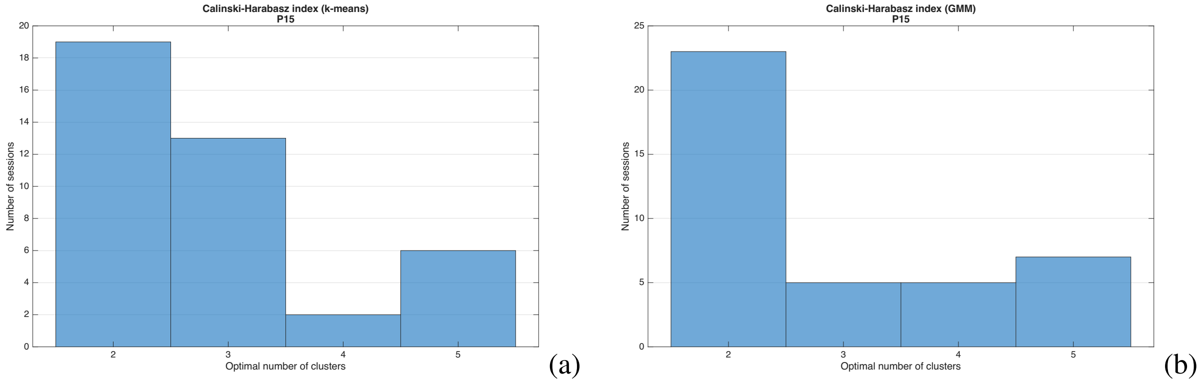

Figure S3. Optimal number of clusters for Patient P15 using **(a)** k-means, **(b)** GMM.

REFERENCES

Bezdek, J. C. (1981). *Pattern Recognition with Fuzzy Objective Function Algorithms*. 1st edition (Springer, Boston, MA). doi:10.1007/978-1-4757-0450-1

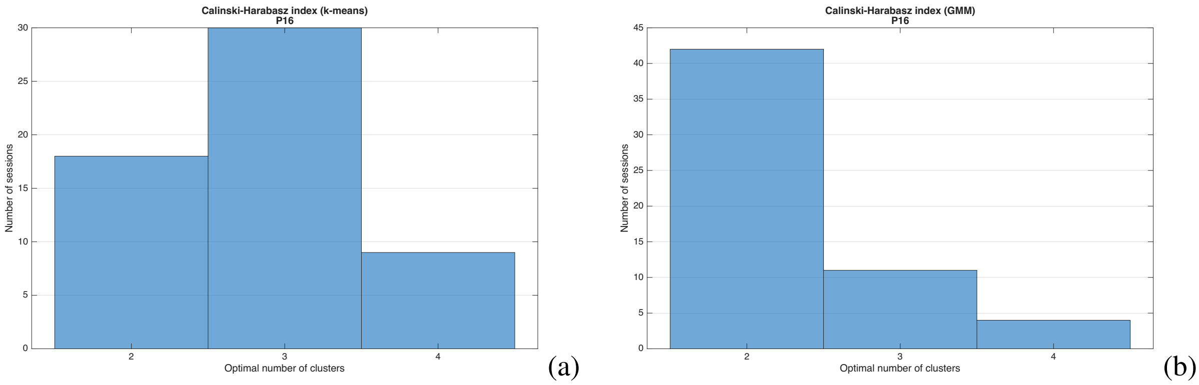

Figure S4. Optimal number of clusters for Patient P16 using **(a)** k-means, **(b)** GMM.

Calinski, T. and Harabasz, J. (1974). A dendrite method for cluster analysis. *Communications in Statistics* 3, 1–27

Ferraro, M. B. and Giordani, P. (2020). Soft clustering. *WIREs Computational Statistics* 12, e1480. doi:10.1002/wics.1480

McLachlan, G. and Peel, D. (2000). *Finite Mixture Models* (John Wiley & Sons, Inc.). doi:10.1002/0471721182
